# Obstacles for self-management practices among diabetes patients: A facility-based study from Coastal South India.

**DOI:** 10.12688/f1000research.138146.1

**Published:** 2023-07-17

**Authors:** Rekha T, Murali Mohan R, Nithin Kumar, Kausthubh Hegde, Bhaskaran Unnikrishnan, Prasanna Mithra, Ramesh Holla, Balanarayana Suma, Aadithya M Rao, Patil Nikitha, Aysha Roushida Sahama M

**Affiliations:** 1 Department of Community Medicine, Kasturba Medical College, Mangalore, Manipal Academy of Higher Education, Manipal, India

**Keywords:** Type 2 Diabetes mellitus, Cross-sectional study, Blood glucose self-monitoring, Socioeconomic status, Diabetes Obstacles Questionnaire-30

## Abstract

Background

The purpose of the study was to assess the obstacles faced by diabetes patients in their self-care and determine the factors associated with these obstacles. The management of diabetes mellitus (DM) extends beyond the clinician’s efforts, with the responsibility of the care also being shared by the patient to achieve better treatment outcomes and prevent complications. Self-care management is the most important part of DM treatment, which includes diet, regular exercise, blood glucose monitoring, medication and foot care.

Methods

A facility-based cross-sectional study was conducted among 107 type 2 DM patients aged >18 years using the Diabetes Obstacles Questionnaire-30. Patients scoring a mean response score >3 were considered to have an obstacle. We included age, gender, socioeconomic status (SES), duration of DM and blood glucose levels as factors for regression analysis and a P value <0.05 was considered to be statistically significant.

Results

A large majority (64.5%, n = 69) of our participants were aged above 55 years and belonged to lower socio-economic status (65.4%, n = 70). Family history of DM was present in 41% (n=44) of the participants. The median duration of DM among the participant was 10 (4 – 7) years.

In our study, the participants faced obstacles for two items in the domains: Support from Friends & Family (mean score: 3.73) and Knowledge of the Disease (mean score: 3.58). A multinominal regression analysis revealed SES was predictive of participants who could not understand information from literature with a P. value of 0.002 (OR: 3.65, CI: 1.60-8.338).

Conclusion

The two major obstacles to self-management practices that were identified were in the domains of Support from Friends and Family, and Knowledge of the Disease. Socioeconomic status was identified to be a predictive factor associated with the participants who are not able to understand information from the literature.

## Introduction

Diabetes mellitus (DM) is a complex, chronic metabolic illness characterized by continuously elevated blood glucose levels which if not controlled will lead to complications related to the eyes, nerves, kidneys, and cardiovascular system, ultimately resulting in death. ^1^ The World Health Organisation (WHO) has estimated that DM was responsible for over 1.5 million deaths in 2019, with 48% of the deaths occurring before 70 years of age. ^2^ Affecting over 537 million people worldwide, the prevalence of DM has been rising over the past 3 decades. ^3^ The rise in prevalence is predominantly reported in low- and middle-income countries, where deaths due to DM have increased by 13% over the past 20 years. ^1^^,^^3^^,^^4^

Accounting for 17% of the total diabetes population, India is often referred to as the ‘Diabetes capital’ of the world. ^5^ India has an estimated 77 million people living with diabetes.

According to the International Diabetes Federation (IDF), India ranks among the top three countries with the highest populations of people with diabetes, along with China, and the USA. ^6^

The situation is made worse by the fact that 25 million people are in the stage of prediabetes, and are at higher risk of developing overt diabetes if their blood glucose is not controlled. The National Noncommunicable Disease Monitoring Survey (NNMS) carried out among adults aged between 18-69 years reported a 9.3% and 24.5% prevalence of DM and impaired fasting blood glucose (IFG) respectively. ^7^ Also, 50% of the population in India is unaware of their diabetes status. A multi-centric study conducted in 2017 reported that 47% of DM cases remain undiagnosed in India, emphasizing the fact that awareness regarding the disease remains poor in the population. ^8^ These undiagnosed cases of DM will end up with complications and burden the health system at various levels of care.

The complications arising from untreated or poorly treated DM are similar for both type 1 and type 2 DM, and make up a large portion of the morbidity and mortality associated with the disease. Diabetes also leads to significant financial implications, with the global economic burden estimated around US $1.31 trillion. ^9^ Effective control and management of diabetes can prevent or delay the progression of the complications and can reduce the morbidity, mortality and the costs associated with this condition. ^10^

Treatment of diabetes is multifactorial, with multiple approaches working synergistically to control hyperglycaemia in the body. Weight loss and intensive lifestyle modifications are initial modalities of treatment, along with pharmacologic methods including drugs of various categories, like metformin, sulfonylureas, glinides, DPP-4 Inhibitors, SGLT-2 inhibitors among others. ^11^

In addition to the various modalities of treatment available, self-management plays a pivotal role in the management of DM and prevention of its complications. Self-management is the regular activities the patients with DM do to control or lessen the effects on their physical health status. ^12^ Self-care in DM is defined as an evolutionary process of knowledge or awareness development by learning to deal with the complexities of DM in a social context. ^13^

Practices focusing on self-care in diabetes include regular monitoring of blood glucose levels, regular physical activity, healthy eating, foot care, and compliance to medication should be given utmost importance. ^13^ Relationships with medical professionals, support from friends and family, knowledge of the disease, and uncertainty about consultation are other important factors that can influence diabetes self-care. ^10^

Self-monitoring of glucose levels provides a quick and easy way for a patient to identify periods of hyperglycaemia and undergo interventions as necessary. It can be used to promote modifications in treatment by a healthcare practitioners and is the most beneficial for those patients with suboptimal control over their glucose levels. ^14^^,^^15^

Diet control is one of the most significant factors that can be used to determine the level of self-care established. ^13^ Weight loss is a cornerstone of the dietary recommendations of the American Diabetes Association, which recommends a lowered caloric intake to successfully reduce obesity and induce weight loss. ^16^ Physical activity and exercise improves the physical and psychological health of the patient and also acts as an adjunct to the weight loss regimen.

Foot care covers a variety of interventions such as foot temperature measurement, use of therapeutic footwear, patient education, inspection and assessment by professionals, with varying impacts. ^17^ Proper foot care practices can lead to reduced development of complications like ulcers and amputations. ^18^

Adherence to the treatment is also critical as missed dosage of the prescribed drugs can lead to elevated glucose levels and development of the complications. It is important to consider perspectives of both the patient and provider perspectives when exploring self-care barriers. Improved self-care behaviour adherence has been linked to increased patient involvement in treatment decisions and satisfaction with provider communication. ^19^ Patient’s knowledge about the disease, his attitude, and perception towards lifestyle modifications and various other aspects of diabetes self-care including treatment adherence and advice is considered to be important drivers for the prevention and control of diabetes. ^13^ Clinicians may further influence the patient’s knowledge and attitude may further be influenced by the clinicians by using effective communication techniques and by having a healthcare system that is well-integrated. ^20^

Although self-care practices play crucial part in the control of DM, the patients find this complex process quite difficult to achieve on a day-to-day basis and face several barriers as they navigate through this approach.

Several factors affect a patient’s ability to monitor blood glucose levels on a day-to-day basis. The studies from other countries have reported obstacles arising from poor communication between the medical practitioner and patients, to limited access to facilities and equipment. ^21^ Another common factor that results in poor management of DM is the inadequate knowledge about the disease or its management either due to ignorance or scarcity of trustworthy sources that provide information regarding the disease.

In developing counties, financial difficulties majorly affect self-care and monitoring. Poor moral support from the family members or the lack of desire to impact a change on patients’ lifestyle also affects their self-care. To prevent the spread of COVID-19, countries resorted to frequent lockdown. This in turn, resulted in decreased access to outpatient care for regular check-ups. Isolation and stress also resulted in mental health issues, and this in turn also acted as barrier to self-management of DM. ^22^

Understanding diabetes and its necessity of long-term management requires self-motivation and support from loved ones. As this disease has no cure and can only be controlled in the long run, it becomes a challenging issue for doctors, community workers and patients to effectively manage the same.

Effective self-management has extended psychological benefits, with decreased anxiety and depression reported among adherent patients. ^23^ Appropriate self-management of diabetes can improve the quality of life, reduce healthcare expenses, and decrease the rate of complications.

Self-management thus plays a crucial part in the control of diabetes and the benefits it provides are immense. It is prudent to identify the barriers and obstacles that may decrease the utilisation of these practices.

Understanding and identifying these challenges at the early stage of treatment is very important to achieve optimal blood glucose control and prevent complications. There is a paucity of literature regarding obstacles to diabetes self-care practices in India. With this background, a cross-sectional study was carried out to identify the obstacles to self-care among type 2 DM patients and the factors associated with the obstacles.

## Methods

### Study area

Mangalore is a major port city in the district of Dakshina Kannada and is one among the largest city in the southern State of Karnataka, with a population of over 6 lakhs. ^24^ With an average literacy rate of 93.7% ^24^ which is higher than the national figure of 85%, the city is considered to be one among the educational hub in South India. It also serves as a major economic and health care centre with a health care index of 62. ^25^

Dakshina Kannada is one among the high burden districts for diabetes with a prevalence of 14.8% and 15.4% among men and women respectively. ^26^

This facility based cross-sectional study was carried out in the hospitals affiliated to Kasturba Medical College, Mangalore.

### Participants

In this cross-sectional study, 107 patients with type 2 DM attending the medicine out-patient department in the hospitals affiliated to Kasturba Medical College, Mangalore were assessed regarding the obstacles for practicing diabetes self-care. All the participants were aged 18 years and above, and were diagnosed with DM for at least a year The study was conducted in the months of November and December, 2019.

The sample size of 107 was calculated based on the assumption that 50% of the DM patients face obstacles when practicing self-care, with an absolute precision of 10%, 95% confidence interval and adding a 10% non-response error. Gender of the participants was documented based on self-report. Gender difference in seeking care was not taken into consideration for the design of the study and reporting of the results, since it was a facility based study and patients were already seeking health care, we assumed that the barriers faced by the patients of either gender would be similar.

### Data collection

The participants were briefed about the purpose and objectives of the study. Written informed consent was obtained from the willing participants, following which the participants were taken to a separate room to ensure privacy, and face-to-face interviews were conducted by one of the authors (interviewer) who were familiarised with the contents of the semi-structured questionnaire, to avoid any bias in interview. The interviews were conducted in the language the respondents were comfortable with and the responses were recorded in a printed questionnaire by the interviewer at the time of interview. The duration of the interview was for 15-20 minutes on average.

The data was collected using a semi-structured questionnaire prepared after extensive review of literature. The questionnaire consisted of the following sections:

Section A: Socio-demographic information of the study participants

Section B: Details regarding the diabetes and self-care practices followed by the participants

Section C: Diabetes Obstacles Questionnaire-30 (DOQ-30) ^10^

Section D: Clinical examination and lab investigations

### Diabetes Obstacle Questionnaire-30

The Diabetes Obstacle Questionnaire-30 is a validated instrument developed by Pilv L, *et al.* ^10^ and consists of 30 items which gives a measure of Diabetes Related Quality of Life in nine subscales. The nine subscales include Relationship with medical professionals, Support from friends and family, Knowledge of the disease, Lifestyle changes, Exercise, Self-monitoring, Uncertainty about a consultation, Medication and Insulin-use among others. Each sub-scale comprises of 2 to 4 items which were assessed using a five-point Likert-scale (i.e., 5-Strongly Agree, 4-Agree, 3-Neutral, 2-Disagree and 1-Strongly Disagree).

The questionnaire was translated to Kannada, a local language, and was pretested and content validated for the language. The socioeconomic status of the participants was assessed using Modified Kuppuswamy’s Scale. ^27^

### Ethical considerations

The study protocol was approved by the Institutional Ethics Committee (IEC) of Kasturba Medical College, Mangalore (IEC number: IECKMCMLR/022/2018).

Permission was obtained from the Medical Superintendent of KMC Hospital, Attavar, Mangalore, and District Medical Officer of Government Wenlock Hospital, Mangalore for conducting the study among the patients.

### Data management and analysis

The collected data was entered in and analysed using IBM SPSS (Statistical Package for Social Sciences) Statistics version 25.0 for Windows (Armonk, NY: IBM Corp). The results were expressed using mean (standard error of mean), median (inter-quartile range) and proportions.

For the purpose of analysis and ease of interpretation, strongly disagree/disagree and strongly agree/agree were stratified into disagree and agree, respectively.

The items in the domains of DOQ-30 were considered an obstacle if the mean response score was 3 or more.

For the items which were identified to be an obstacle, multinomial logistic regression (MLR) analysis was applied taking neutral as reference modality to find out the factors responsible for the respective obstacle. Regression analysis with age, socioeconomic status (SES), duration of DM and blood glucose level as possible factors was done to find any association. Odds ratio and corresponding confidence interval were reported and P < 0.05 was considered as statistically significant association. The missing data, if any, were not considered for the analysis by deleting the variable response listwise.

## Results

A total of 107 type 2 DM patients were included in the study. A large majority (64.5%, n = 69) of our participants were aged above 55 years, with the mean age of 60.3 (±10.9) years. A higher proportion (72%, n = 77) of the participants were males and belonged to lower socio-economic status (65.4%, n = 70). Family history of DM was present in 41% (n=44) of the participants.

The median duration of DM among the participant was 10 (4–7) years. About 58% (n = 62) of the participants had at least one comorbidity, with hypertension being the most common (82.2%, n = 51). The general information of the study participants is given in Table 1. The full dataset can be found under *Underlying data.* ^46^

**Table 1.** Study participants information (N = 107).

Variables	n (%)
**Age group (in years)**	
≤45	11(10.3)
46–55	27(25.2)
>55	69(64.5)
**Gender**	
Male	77(72.0)
Female	30(28.0)
**Religion**	
Hindu	84(78.5)
Christian	14(13.1)
Muslim	09(08.4)
**Socioeconomic status** *	
Upper	02(01.9)
Upper middle	11(10.3)
Lower middle	24(22.4)
Upper lower	66(61.7)
Lower	04(03.7)
**Family history of diabetes**	
Present	44(41.1)
Absent	63(58.9)
**Comorbidity**	
Present **	62(57.9)
Absent	45(42.1)
**Smoking status**	
Never	77(72.0)
Past	22(20.6)
Current	08(07.5)
**Alcohol status**	
Never	72(67.3)
Past	26(24.3)
Current	09(08.4)

* Modified Kuppuswamy scale, 2017.

** Hypertension – 51 (47.6%).

The obstacles for diabetes self-care as assessed using DOQ-30 is depicted in Table 2. The mean response score for most of the items under various domains was less than 3.

**Table 2.** Obstacles for self-care among the study participants - findings of Diabetes Obstacles Questionnaire-30 (N = 107).

Subscales	Mean (SE of mean)	Agree n (%)
**Relationship with medical professionals**		
I am not assisted in setting realistic targets to change my lifestyle	2.35(0.13)	32(29.9)
I have not been told what to expect from my treatment	2.41(0.13)	30(28.0)
The good and bad aspects of each choice have not been discussed with me	2.66(0.13)	31(29.0)
Treatment alternatives are not explained to me	2.67(0.14)	38(35.5)
**Support from friends and family**		
I feel I get little support from my family	1.92(0.12)	20(18.7)
I feel I get little support from my friends	2.11(0.13)	25(23.4)
I feel very alone with my diabetes	2.04(0.11)	15(14.0)
I would manage my diabetes much better if I had encouragement socially	3.73(0.12)	79(73.8)
**Knowledge of the disease**		
I do not know as much as I need to know about the consequences of having diabetes	2.73(0.12)	37(34.6)
I do not know enough about the treatment of diabetes	2.71(0.12)	35(32.7)
I do not know as much as I need to know to manage my diabetes	2.82(0.12)	38(35.5)
I have difficulty understanding information from literature	3.04(0.12)	45(42.1)
**Lifestyle changes**		
My diabetes has placed a strain on my personal relationships	2.88(0.13)	40(37.4)
Changes in my diet have put a strain on my family	2.60(0.13)	31(29.0)
I feel resentful that I am obliged to change my eating habits	2.82(0.12)	34(31.8)
My diabetic diet spoils my social life	2.83(0.13)	35(32.7)
**Exercising**		
I have not found an exercise I enjoy	2.93(0.11)	30(28.0)
I lack the motivation to exercise	2.90(0.11)	33(30.8)
I am unable to fit exercise into my lifestyle	2.85(0.10)	28(26.2)
I am unable to afford the cost of exercising on a regular basis	2.67(0.10)	19(17.8)
**Self-monitoring**		
Self-monitoring makes me feel frustrated	2.73(0.07)	6(5.6)
I find it too uncomfortable to self-monitor	2.75(0.07)	9(8.4)
I find it extremely hard to test when I am busy	2.70(0.06)	3(2.8)
Self-monitoring makes me fearful of a high reading	2.74(0.06)	6(5.6)
**Uncertainty about a consultation**		
The way that I was told I had diabetes made me feel afraid	2.87(0.14)	49(45.8)
I feel a sense of helplessness when I am consulting with the nurses	2.04(0.09)	7(06.5)
**Medication**		
I do not feel I am being prescribed a medication dose that is right for me	1.93(0.12)	21(19.6)
I do not feel I am being prescribed a medication that is right for me	1.94(0.12)	22(20.6)
**Insulin use**		
Using insulin makes life too complicated	2.73(0.07)	10(09.3)
Using insulin means my diabetes is getting worse	2.69(0.07)	07(06.5)

However, in one item each from the domains of Support from Friends and Family, and Knowledge of the disease, the participants scored more than the average cut-off of 3, suggesting an obstacle.

The mean response score for item, ‘I would manage my diabetes much better if I had encouragement socially’ (item one) under the domain, Support from Friends and Family, was 3.73 (0.12 SEM) with 79 (73.8%) participants agreeing that social support is important for diabetes control. The mean of response to the item, ‘I have difficulty understanding information from literature’ (item two) under the domain, Knowledge of the Disease was 3.58 (0.09 SEM) with 66 (61.7%) participants agreeing that they were unable to understand the information related to diabetes from the literature.

Multinomial logistic regression (MLR) was applied on the two items identified to be obstacles for self-care to find out the significant factors associated with them.

About 74% (n = 79) of participants stated that item one was an obstacle for self-care practice, while 7.47% (n = 8) of them were neutral about their response to this statement. MLR analysis with neutral as the reference category revealed no predictive factors for this obstacle.

Item two was an obstacle for 61.68% (n = 66) of the participants, while 23.3% (n = 25) were neutral about their response. MLR analysis revealed socioeconomic status is a strong predictor of this obstacle with P. value of 0.002 (odds ratio: 3.65 and confidence interval: 1.60-8.33). The other factors were statistically insignificant. This data is summarised in Table 3. The data of some of the examination findings (height, weight, BMI) and lab values (HbA1c and blood cholesterol levels) were missing. Those parameters were not considered for analysis in our study.

**Table 3.** Factors associated with obstacles for self-care practices among diabetes patients (N = 107).

Factors	Agree		Disagree	
	Adjusted odds ratio (CI)	P value	Adjusted odds ratio (CI)	P value
**Item 1: I would manage my diabetes much better if I had encouragement socially**				
Age	0.96 (0.89-1.03)	0.329	0.99 (0.912-1.07)	0.835
Socioeconomic status	1.71 (0.71-4.09)	0.226	2.22 (0.764-6.48)	0.143
Diabetes duration	1.04 (0.95-1.15)	0.348	1.00 (0.899-1.12)	0.916
Random blood glucose	0.99 (0.98-1.01)	0.881	1.00 (0.989-1.024)	0.496
**Item 2: I have difficulty understanding information from literature**				
Age	1.00 (0.95-1.05)	0.807	1.01 (0.92-1.09)	0.812
Socioeconomic status	3.65 (1.60-8.33)	0.002	0.23 (0.08-0.64)	0.005
Diabetes duration	1.01 (0.94-1.07)	0.761	1.01 (0.92-1.10)	0.825
Random blood glucose	1.00 (0.99-1.01)	0.783	1.00 (0.99-1.02)	0.470

## Discussion

In this facility based cross-sectional study, 107 type 2 diabetes patients were assessed about the obstacles for diabetes self-care practices using the Diabetes Obstacles Questionnaire-30 (DOQ-30). ^10^ Very few studies have been conducted to understand the barriers for self-management practices and to determine possible predictive factors for these obstacles among the DM patients.

Among the 30 items across 9 domains of self-care, only 2 items were considered as obstacles by the participants in our study. The lack of social encouragement for better management of disease and difficulty in understanding the literature about the disease were agreed upon by the participants to be a challenge for practicing self-care.

Lack of social support as a barrier for diabetes self-management was reported by a multi-national study, where effective social support system with constant motivation and encouragement from friends and family members were identified as enablers for self-management. ^28^ The importance of social support in diabetes has been documented in multiple studies, where strong social support leads to a better degree of self-management and overall positive outcomes. ^29^^,^^30^ A systematic review and meta-analysis conducted by Youngshin *et al.* ^31^ found social support to be significantly associated with self–management, with the strongest effect on regular glucose monitoring. On the contrary, lack of support and isolation, for example a patient living alone, is associated with poor self-management, including a reluctance to use insulin, medicines, and irregular testing of blood glucose levels. They are also more likely to not follow any diet plan to avoid the stigma and embarrassment associated with diabetes. ^32^ A lack of support from family and friends can directly lead to poorer psychological wellbeing, improper or less than adequate self-management, and ultimately lead to suboptimal glycaemic control and poor outcomes for the patient.

Difficulty in understanding diabetes related literature was also identified as an obstacle in our study. Poor level of knowledge among patients with chronic diseases, especially diabetes is well documented, with low level of health literacy being reported between 15 to 40% of the population. ^33^ Various studies from USA and Japan have documented that low health literacy is associated with poor diabetes knowledge, which can in turn affect the practice of diabetes self-care. ^34^^–^^36^

The role of education in diabetes self-care is well documented with various studies reporting a correlation between lower educational status of the patient and poor self-management practices. ^37^^–^^39^ A study by Aziz, *et al.*, ^18^ reported a correlation between education of the patient and foot care practices, along with a decreased rate of amputations associated with diabetes. A similar study conducted by Chai *et al.* ^40^ showed a significantly increased psychological health and decreased glucose levels after six months following self-management education. An integrative review by Olesen *et al.* ^41^ found a patient centred approach to diabetes self-management education to have a noticeable impact in improving the self-management, leading to better haemoglobin A1c levels, as well as the quality of life of the patients.

Several studies have reported an adequate control of blood glucose levels and improvement in morbidity and mortality if the patients follow self-management behaviours such as blood glucose monitoring, nutrition, exercise, medication, and foot care. ^18^^,^^37^^–^^41^ Diabetes education is thus very important and should be an integral part of every diabetes control program.

Social support and education have been identified to work synergistically to bring about improvement in clinical outcomes in a diabetes patient. ^12^

The other primary objective of our study was to identify the factors that may be predictive of the obstacles for the self-care management among diabetes patients. We considered two statements from DOQ-30: ‘I would manage my diabetes much better if I had encouragement socially’ and ‘I have difficulty understanding information from literature’, which were reported to be the obstacles faced by the study participants and we tried to predict the factors for these obstacles.

MLR analysis revealed socioeconomic status (OR – 3.65, CI – 1.60-8.33 and P – 0.002) to be significantly associated with participants not being able to understand the diabetes related literature. No factors were found to be significantly associated with lack of social support for diabetes management among the participants.

Diabetes self-care is a complex process which involves dietary modifications, regular exercising, regular blood glucose monitoring and compliance to treatment, all of which work synergistically to achieve an optimum blood glucose level. Older diabetes patients have a unique set of problems which make practicing diabetes self-care challenging. Decreased physical and cognitive abilities, presence of comorbidities, and complications, polypharmacy, absence of support system, change in dietary habits, and depression associated with chronic diseases will hamper the diabetes self-care behaviour. ^42^ Increased age could possibly be a predictor for hesitancy and reluctance in obtaining information and a difficulty in comprehension.

Socio-economic status is a multidimensional factor, which is a combination of educational, economic and occupational status. It is linked with Social Determinants of Health (SDOH) which includes accessibility to quality health care, good nutrition, transportation, housing, and social engagement, all of which can affect disease management. Low SES indicates a lower level of education. In our study, SES was found to be a predictive factor for patients not being able to understand information regarding diabetes from literature. All levels of SES have been consistently reported to be a strong predictor for disease onset and progression for many chronic diseases, including diabetes. ^43^

Patients belonging to lower SES are more likely to develop diabetes, poor self-care management, experience complication, than those belonging to higher SES *.* ^44^^,^^45^

The obstacles of diabetes self-care faced by the participants in our study were mainly due to their poor education and lower economic status. Based on the results of the DOQ-30, the majority of the patients did not have any problem following a diet or an exercise pattern as they felt that these lifestyle modifications were necessary to lead a longer and better life. The healthy relationship between the participants and healthcare providers likely ensured better information exchange to manage their diabetes in an efficient manner.

### Limitations

The results of this facility-based investigation cannot be generalised to the broader population. The domain of self-monitoring was difficult to assess since the majority of the participants in our study belonged to low SES and did not have provision for self-monitoring of blood glucose. Also, the domain of Insulin use was also difficult to assess since the majority of our study participants were not prescribed insulin.

## Conclusions and recommendations

In our study, the two major obstacles for self-management practices that were identified were in the domains of Support from Friends and Family, and Knowledge of the Disease. Socioeconomic status was identified to be a predictive factor associated with the participants who are not able to understand information from the literature. Self-management is an integral component of the treatment plan of diabetes and can lead to more positive outcomes. Targeting these barriers as identified in our study could improve self-management. Having a strong social support network could be advised and encouraged. An increased focus on patient education and awareness both in primary care as well as awareness programs could lead to decreased rate of complications, and an increased quality of life for patients moving forward.

## Data Availability

OSF: “Obstacles for Self-Management Practices Among Diabetes Patients - A Facility-based Study from Coastal South India.” https://doi.org/10.17605/OSF.IO/9EN45. ^46^ This project contains the following underlying data:
- Final.xlsx [raw data] This project contains the following extended data:
- Key.xlsx [This Microsoft Excel Spreadsheet has the key to interpret the data in the file named Final.xlsx] Data are available under the terms of the Creative Commons Zero “No rights reserved” data waiver (CC0 1.0 Public domain dedication).
