# Supplementary material for: Obstacles for self-management practices among diabetes patients: A facility-based study from Coastal South India
Source: F1000Res. 2023 Jul 17;12:839. [Version 1] doi: 10.12688/f1000research.138146.1 (PMC11923534; doi:10.12688/f1000research.138146.1)
Supplement: Peer review history

# Reviewer report: Reviewer response for version 1

**DOI:** 10.5256/f1000research.151323.r273975

**Published:** 2024-09-26

**Authors:** Ganesh Kumar Saya

**Affiliations:** 1 Department of Preventive and Social Medicine, Jawaharlal Institute of Postgraduate Medical Education and Research (JIPMER), Puducherry, Tamil Nadu, India

This is an important public health issue due to the high problem burden of diabetes. However the following points may be clarified:

1. Since this study is done in a tertiary care hospital, the original residential status of the participant details may be mentioned in methods section. 
2. Whether there are similar studies by using the same questionnaire? If so, it may be applied for minimal sample size calculation.
3. Whether details of accepted standard self care practices may be elaborated in methods section as mentioned in section B of data collection? If self management practice also convey the same meaning, same words may be used.

Is the work clearly and accurately presented and does it cite the current literature?

Yes

If applicable, is the statistical analysis and its interpretation appropriate?

Yes

Are all the source data underlying the results available to ensure full reproducibility?

Yes

Is the study design appropriate and is the work technically sound?

Yes

Are the conclusions drawn adequately supported by the results?

Yes

Are sufficient details of methods and analysis provided to allow replication by others?

Yes

Reviewer Expertise:

Maternal and Child health, disability

I confirm that I have read this submission and believe that I have an appropriate level of expertise to confirm that it is of an acceptable scientific standard.

# Response

**Published:** 2024-11-28

**Authors:** Nithin Kumar

**Affiliations:** Kasturba Medical College Hospital, India

1. **Since this study is done in a tertiary care hospital, the original residential status of the participant details may be mentioned in methods section**. 

Response: Thank you, sir, for the suggestion. The details have been incorporated in the manuscript.

1. **Whether there are similar studies by using the same questionnaire? If so, it may be applied for minimal sample size calculation.**

Response: During our review of literature, we couldn’t find any Indian studies where self-care practices was assessed using the Diabetes Obstacles Questionnaire-30 (DOQ-30). Few of the studies had used Summary of Diabetes Self-care Activities (SDSCA) scale. Hence we went by the assumption for sample size calculation.  

1. **Whether details of accepted standard self-care practices may be elaborated in methods section as mentioned in section B of data collection? If self-management practice also conveys the same meaning, same words may be used**

Response: The participants were asked about diabetes self-care practices under the domains of diet, exercise, blood glucose monitoring, medication compliance, and foot care. They were also asked if they had any self-reported obstacles to following these practices during the preceding week of the study.

 Diabetes self-management and Diabetes self-care practices are the same.
